# Advances in Small Molecular Agents against Oral Cancer

**DOI:** 10.3390/molecules29071594

**Published:** 2024-04-03

**Authors:** Kai Wei, Weiru Zhu, Yanan Kou, Xinhua Zheng, Yunyun Zheng

**Affiliations:** 1 Medical School, Pingdingshan University, Pingdingshan 467000, China; weikai1987@126.com (K.W.); 211041021@e.pdsu.edu.cn (W.Z.); xinhuazheng@pdsu.edu.cn (X.Z.); 2 Affiliated Stomatology Hospital, Pingdingshan University, Pingdingshan 467000, China

**Keywords:** oral squamous cell carcinoma, anti-proliferative, signalling pathway, cell proliferation, cell apoptosis

## Abstract

Oral cancer is a common malignancy with a high mortality rate. Although surgery is the best treatment option for patients with cancer, this approach is ineffective for advanced metastases. Molecular agents are irreplaceable in preventing and treating distant metastases. This review aims to summarise the molecular agents used for the treatment of oral cancer in the last decade and describe their sources and curative effects. These agents are classified into phenols, isothiocyanates, anthraquinones, statins, flavonoids, terpenoids, and steroids. The mechanisms of action of these agents include regulating the expression of cell signalling pathways and related proteases to affect the proliferation, autophagy, migration, apoptosis, and other biological aspects of oral cancer cells. This paper may serve as a reference for subsequent studies on the treatment of oral cancer.

## 1. Introduction

Oral diseases are a global public health problem, affecting the health and quality of life of 3–5 billion people [1,2,3]. If untreated, they may even lead can to systemic diseases [4,5]. Thus, many studies have focused on the aetiology, pathogenesis, and therapeutic regimens of oral cancer [6,7,8].

Oral cancer often arises from pre-existing white spots and oral submucosal fibrosis, and its incidence has increased with the increasing consumption of carcinogens, such as tobacco and alcohol [9,10,11]. The types of oral cancer include cancer of the palate, tongue, floor of the mouth, lip, buccal mucosa, etc., and vary according to the location of the infected cancer cells [12]. Physiological disorders are caused by the disease itself or traditional treatment, such as pain, paraesthesia, dysphagia, dysphagia, infection, ulceration, maxillofacial deformity, and other complications. Oral cancers have a significant genetic diversity, and these subgroups include p53-independent tumours, subtypes with multiple tumour suppressor l (MTS1), oral leucoplakia, etc. [13]. Tongue cancer is mainly caused by cell cycle-related gene cyclin D1 changes [14]. These oncogenes influence the clinicopathological features of oral squamous cell carcinoma, including poor tumour differentiation, lymph node involvement, and poor survival [15]. Oral squamous cell carcinoma (OSCC) is the leading cause of cancer-related deaths, and its incidence and mortality are increasing considerably annually [16,17,18]. Despite advances in diagnostic imaging, surgery, radiation, and chemotherapy, oral cancer is often diagnosed at a later stage of disease development, leading to poor prognosis and high mortality. Moreover, many patients with oral cancer are resistant to standard treatments owing to heterogeneity within the tumour or a genetic mutation which occurs during treatment, resulting in the high recurrence rate of this disease [19,20,21]. Oral cancer metastasises to various tissues or organs of the body through the lymphatic system or blood, and generally has no specific location, but it is more likely to metastasise to the head and neck area [20]. Thus, the development of safe and reliable drugs is crucial for the effective treatment of oral cancer. Different cell death pathways, immunotherapy, and the targeted inhibition of tumour cells have been explored for the treatment of malignant tumours [12,22]. Additionally, target discovery and validation are the key steps in developing molecular agents for the treatment of oral cancer. Multiple signalling pathways are involved in the progression of oral cancer, such as Toll-like receptor 4 signalling (TLR4), phosphoinositide 3-kinase (PI3K) pathway, janus kinase (JAK)–signal transduction and activator of transcription (STAT) pathway, etc. [23]. The study of Kenison et al. indicates that it is of great significance to develop immune checkpoint inhibitors targeting aromatic hydrocarbon receptors for oral cancer immunotherapy [24]. Current research is focused on discovering new targets of oral cancer drugs and in verifying targets of traditional drugs [25].

Various oral cancer drugs have been launched with the recent rise in the occurrence, development, and diagnosis, of oral cancer; further, the continuous development of clinical trials on molecular targeted therapies has accelerated this process. This review summarises the molecular agents used to treat oral cancer and their mechanisms of action, pharmacological advantages, and development strategies. It also discusses research progress in oral cancer drugs and candidates. This paper may serve as a reference for designing novel oral cancer drugs with simple structures and good efficacy.

## 2. Polyphenols

**Figure 1:**
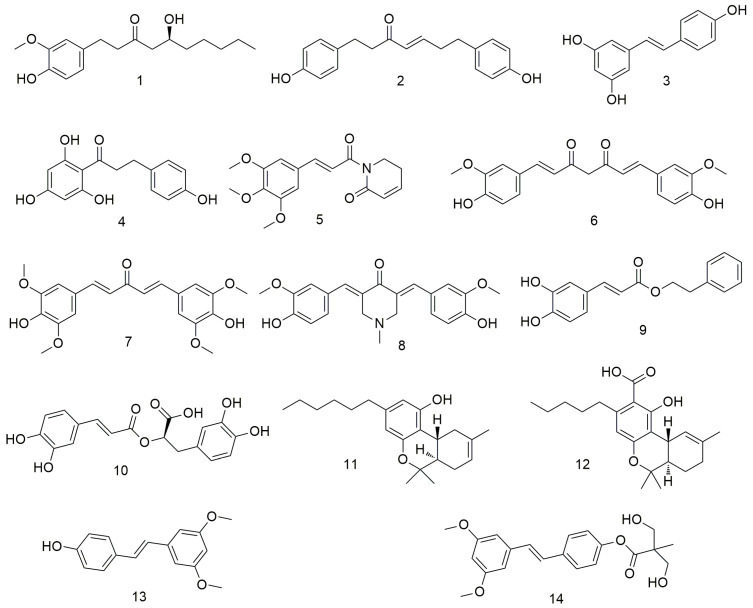
Polyphenol agents.

Natural polyphenols (Figure 1) have emerged as promising chemopreventive and anti-cancer agents [26,27,28]. They exert anti-proliferative, anti-metastatic, and pro-apoptotic effects on tumour cells. Natural polyphenols can function synergistically with chemotherapy drugs to overcome drug resistance. Considering the anti-cancer, anti-metastatic, and chemopreventive effects of natural polyphenols on oral cancer, several researchers investigated the mechanisms of action of these agents [29]. Kapoor et al. [30] found that [6]-gingerol (**1**) can significantly inhibit the proliferation of oral cancer cells (OCCs) by inducing apoptosis and G2/M phase arrest. 6-Gingerol can also inhibit OCC migration and invasion by regulating N-cadherin and vimentin, inducing AMPK activation in Ca9-22 cells, and inhibiting the AKT/mTOR signalling pathway. Liu et al. [31] found that platyphyllenone (**2**) induces OCC autophagy and apoptosis by regulating the serine/threonine protein kinase B (AKT) and c-Jun N-terminal kinase (JNK) pathways. Resveratrol (**3**) inhibits OCC proliferation by inhibiting the transactivation of the element binding protein 1 (SREBP1), subsequently down-regulating the expression of epidermal fatty acid-binding protein (E-FABP), blocking the proliferation of Ca9-22 cells, and finally inducing autophagy [32,33]. Yang et al. [34] found that phloretin (**4**) exerts anti-proliferative activity against human OCC through reactive oxygen species (ROS)-mediated apoptosis and G0/G1 phase arrest. Piperlongumine (PL, **5**) inhibits the production of tumour necrosis factor-α (TNF-α) and interleukin-6 (IL-6) and the activation of nuclear factor-κB (NF-κB) in pro-inflammatory response [35,36]. Moreover, PL prevents plaque formation, thereby inhibiting the development of malignant phenotypes and the formation of tumour stem cells [35]. At the molecular level, in vitro studies have shown that curcumin (CUR, **6**) suppresses OCC growth by inhibiting SCC-25 cell proliferation and inducing G2/M phase arrest in a dose-dependent manner [37]. A novel synthetic CUR analogue, GO-Y078 (**7**), induces caspase-mediated apoptosis in OCC by up-regulating apoptosis regulatory proteins SMAC/DIABLO and haem oxygenase (HO)-1 [38]. Semlali et al. [39] demonstrated that curcumin analogue (PAC, **8**) dose-dependently inhibits the proliferation of OCC by disrupting cell cycle distribution, down-regulating the expression of oncogenes (cyclin D1) and cyclin-dependent kinase inhibitors (p21WAF1), and increasing the apoptosis, autophagy, and oxidative stress of OCC. Caffeic acid phenethyl ester (CAPE, **9**) dose-dependently inhibits the proliferation of TW2.6 cells by up-regulating the expression of Bax and Puma, activating the Bax protein, and causing conformational changes, mitochondrial translocation, and oligomerisation [40]. Rosmarinic acid (**10**) exerts anti-cancer effects on different human cancer cell lines by inducing apoptosis and G2/M phase arrest, causing endoplasmic reticulum (ER) stress and decreasing the migration potential of cancer cells in a concentration-dependent manner [41,42]. Delta-8- and delta-9-tetrahydrocannabinol (**11** and **12**) inhibits the growth of OCC through various mechanisms, such as inhibiting the expression of epithelial–mesenchymal transition (EMT) markers (such as E-cadherin), reducing the production of ROS, and increasing the expression of glutathione and glutathione [43]. Yang et al. [44] confirmed that pterostilbene (**14**) inhibits the growth of SAS and OECM-1 cell lines and induces autophagy by inhibiting Akt, p38, and extracellular signal-regulated kinase ½ (ERK1/2) and activating the c-Jun N-terminal kinase (JNK) pathways. Huang et al. [45] designed and synthesised a series of bis(hydroxymethyl)propionate analogue prodrugs using natural rosewood stilbene as the lead compound. They screened the anti-proliferative effects of all derivatives on cisplatin-resistant oral squamous cells (CARs) and found that several compounds show stronger antitumour activities than rosewood stilbene and resveratrol.

**Figure 2:**
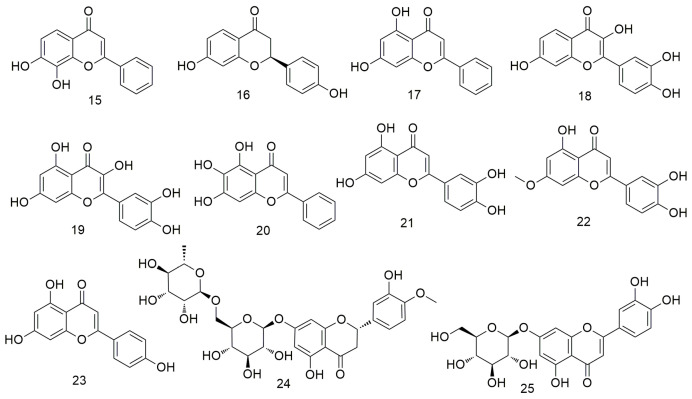
Flavonoid agents.

Flavonoids (Figure 2) are found in plants, including vegetables, fruits, and other foods. These agents prevent the carcinogenesis and proliferation of tumours via various mechanisms, such as regulating the apoptosis and autophagy pathways and causing cell cycle arrest [46,47,48]. 7,8-Dihydroxyflavone (**15**) can induce the apoptosis of OCC by inducing G-phase arrest in OSCC cells and down-regulating specificity protein 1 (Sp1) levels in HN22 and HSC4 cells, indicating that it plays an important antitumour role in OSCC [49]. Liquiritigenin (LQ, **16**) is inactivated via the PI3K/AKT/mTOR pathway, which largely limits tumour growth and enhances apoptosis and autophagy, thereby inhibiting the progression of OCC. In addition, LQ inhibits AKT phosphorylation in tumour tissues [50]. Chrysin (**17**) regulates the apoptosis and autophagy of MC3 cells by inducing MAPK/extracellular signalling, reducing the activity of human mucoepidermoid carcinoma MC3 OCC, and causing morphological changes in MC3 cells [51]. Fisetin (3,3-,4-,7-tetrahydroxyflavone, **18**) is a naturally occurring flavonoid with antioxidant, anti-inflammatory, and anti-cancer properties [52]. This flavonoid enhances the apoptosis of Ca9-22 cells at the human tongue scale through the mitochondrial pathway and inhibition of autophagy. In addition, it can cause cell cycle arrest by disrupting Wnt, mTOR, and NF-xB signals and preventing the invasion and migration of cancer cells. Quercetin (**19**) can cause mitochondrial dysfunction and inhibit the viability, migration, and invasion of OCC via the mitochondrial apoptosis pathway [53,54]. Baicalein (**20**) induces the apoptosis, causes the GO/G1 phase arrest, and reduces the NF-κB activity of OSCC cells. In addition, baicalein inhibits the proliferation of OSCC in vivo and in vitro by down-regulating the relative mRNA levels of the transcription factors Sp1, p65, and p50 [55]. Tu et al. [56] found that luteolin (**21**) combined with radiotherapy reduces the tumourigenicity of OCSC by inactivating the IL-6/STAT3 signalling pathway. Moreover, luteolin treatment reduces the proliferation and self-renewal ability of enriched OCSCs. Huang et al. [57] reported that hydroxygenkwanin (**22**) inhibits cell cycle, cell colony formation, and cell motility by activating p21 and the intrinsic apoptosis pathway. Moreover, apigenin (**23**) can induce the apoptosis of tongue and oral carcinoma-derived cell line SCC-25 and regulate the expression of cyclin D and E, inactivation of cyclin dependent kinase 1 (CDK1), and cell cycle arrest at the G0/G and G2/M phases [58]. Hesperidin (**24**) exerts anti-cancer effects on OCC by inactivating transcriptional actvator 1 (STAT1) and STAT3 signalling molecules and inhibiting programmed cell death 1 ligand 1 (PD-L1) expression [59].Velmurugan et al. [60] demonstrated for the first time that luteosin-7-O-glucoside (**25**) inhibits the invasion and migration of OCC by regulating matrix metalloproteinase-2 (MMP-2) expression and the extracellular signal-regulated kinase pathway and significantly reduces the metastasis of oral cancer by alleviating the P38-induced increase in MMP-2 expression.

## 3. Isothiocyanates

**Figure 3:**
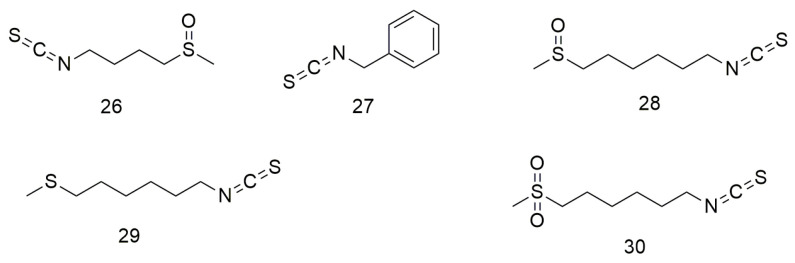
Isothiocyanate agents.

Isothiocyanates (Figure 3) are natural phytochemical compounds derived from plants, such as broccoli, cabbage, papaya, and wasabi, which demonstrate many biological effects, including neuroprotective, anti-inflammatory, and anti-cancer effects. Tsai et al. [61] reported that cathepsin S inhibitors can be used to prevent or delay cancer metastasis. Chen et al. [62] observed that sulforaphane (**26**) reduces the motility and aggressiveness of SCC-9 and SCC-14 cells by decreasing the expression of cathepsin S and inhibits the migration of OCC by regulating the expression of cathepsin S and its downstream target LC3. Varadarajan et al. [63] found that benzyl isothiocyanate (**27**) shows anti-cancer effects on the SCC-25 cell line through G2/M phase blockade and apoptosis induction. 6-MITC (**28**), a wasabi compound, can enhance the sensitivity of OCC cells to the growth inhibitory effect of anti-cancer drugs [64]. Furthermore, 6-MITC and its derivatives 17,447 (**29**) and 17,557 (**30**) inhibit OCC growth in a dose-dependent manner [64].

## 4. Quinones

**Figure 4:**
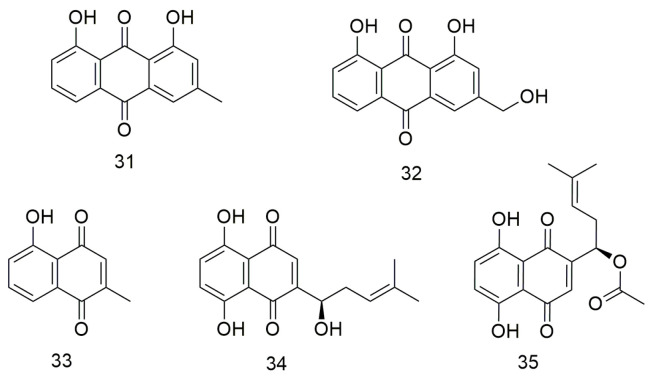
Anthraquinone agents.

Anthraquinones (Figure 4) are a class of quinone compounds that can occur naturally or synthesised artificially. These drugs have various effects, including haemostatic, anti-bacterial, and antitumour. Hsu et al. [65] demonstrated that chrysophanol (**31**) inhibits the proliferation and metastasis and increases the apoptosis of FaDu and SAS cell lines by promoting ROS production and cell cycle G1 arrest. Meanwhile, aloe emodin (**32**) reduces the viability of SCC15 cells and induces apoptosis by regulating the expression of caspase-3/9 [66]. Lin et al. [67] showed that plumbagin (**33**) reduces the viability of CR-SAS cells and induces apoptosis. In addition, plumbagin increases ROS production, leading to mitochondrial dysfunction and ER stress. Animal experiments have also been conducted to demonstrate the in vivo anti-cancer effects of plumbagin on drug-resistant OCC. Shikonin (**34**) enhances the sensitivity of OCC cells to cisplatin. It also inhibits the activity and malignant proliferation of OCC by down-regulating the expression of β-catenin [68]. Acetylshikonin (**35**) significantly inhibits the invasion of YD10B OCC with porphyrin gingival infection by inhibiting IL-8- and IL-8-dependent MMP release [69]. Acetylshikonin (**35**) enhances the phosphorylation of JNK and p38 MAPK via ROS production and triggers apoptosis in Ca9-22 cells [70]. Therefore, acetylshikonin is a strong candidate for a selective chemotherapeutic agent for the treatment of OSCC.

## 5. Statins

**Figure 5:**
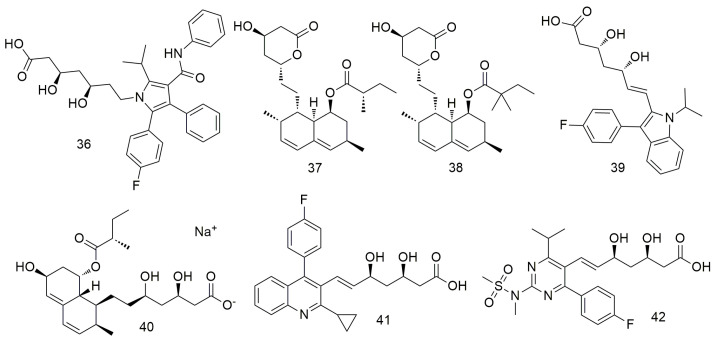
Statin agents.

Statins (Figure 5) inhibit cholesterol biosynthesis by blocking the activity of 3-hydroxy-3-methylglutaryl-CoA (HMG-CoA) reductase and preventing the conversion of HMG-CoA to methanate [71,72,73]. Atorvastatin (**36**) suppresses NADPH oxidase activity and ROS formation by inhibiting Racl activity and induces angiogenesis by increasing VEGF-A expression after ROS formation [74]. In addition, atorvastatin reportedly inhibits the growth of oral tumours by reducing cell migration. This drug creates a toxic microenvironment and inhibits the metastasis of oral squamous cancer cells by increasing intracellular oxidative stress [74]. Lovastatin (**37**) and simvastatin (**38**) inhibit the proliferation of tumour cells by enhancing the response of PD-1 ICB and inducing T cells to kill tumour cells [75]. Combined treatment with daily oral simvastatin (**38**) or lovastatin and PD-1 blocking enhances tumour control and prolongs survival, suggesting that statins may enhance the response to PD-1 checkpoint blocking and other HNSCC immunotherapies [75]. Huang et al. [76] found that statin (**37**–**42**) use significantly decreases the incidence of OCSCC among betel nut chewers.

## 6. Terpenoids and Steroids

**Figure 6:**
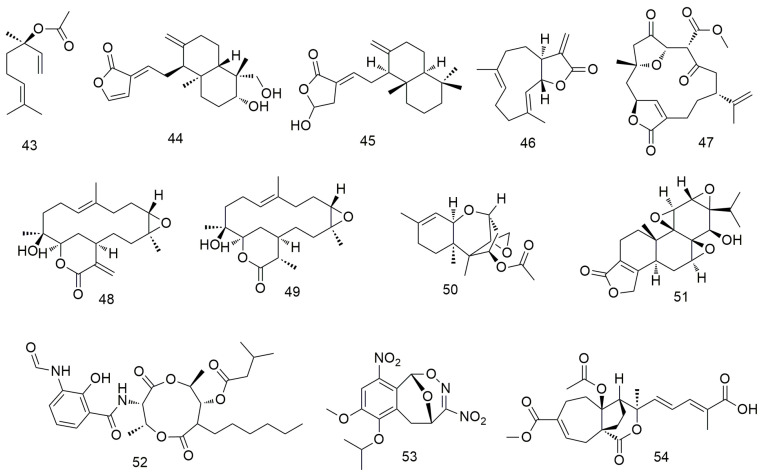
Terpenoid agents.

Zhang et al. [77] showed that linalool (**43**, Figure 6) monoterpene exerts its antitumour effect by reducing the mitochondrial membrane potential and inhibiting the cell cycle and PI3K/AKT signalling pathway. Dehydroandrographolide (**44**) induces autophagy in human OCC by regulating the expression of p53, activating JNK1/2 and inhibiting Akt and p38 expression [78]. It can also effectively inhibit tumour formation in vivo in xenotransplantation models of oral cancer. Coronarin D (**45**) can significantly reduce cancer cell viability by increasing the loss of mitochondrial membrane potential and the expression of death receptors, resulting in the activation of caspase-3/8/9 [79]. It also induces the apoptosis of human SCC-9 and SAS cells by causing G2/M phase arrest, decreasing the activation of ERK1/2, p-38, and AKT, and increasing the activation of JNK1/2. Costunolide (**46**) triggers cell apoptosis by inhibiting AKT activity and significantly promoting ROS production [80]. In addition, an in vivo mouse model analysis showed that costunolide strongly inhibits the growth of cell-derived xenograft oral cancer. 4-Carbomethoxyl-10-epigyrosanoldie E (**47**) induces ROS production in OCC, thereby initiating multiple cellular pathways, including ER stress and mitochondria-induced apoptotic pathway dysfunction, ultimately leading to autophagy [81]. Sinularin (**48**) exerts oxidative stress-mediated anti-proliferative, G2/M-blocking, and apoptotic effects on OCC and is associated with ROS production, making it a potential marine drug against oral cancer [82]. Yang et al. [83] confirmed that dihydrosinularin (**49**) exerts its anti-proliferative effect on OCC by inducing apoptosis, double-strand breaks, and DNA oxidative damage without causing cytotoxicity to non-malignant oral cells. Trichodermin (**50**) inhibits the migration and invasion of OSCC Ca922 and HSC-3 cells by down-regulating the expression of MMP-9. In addition, trichodermin can reduce the mitochondrial membrane potential and mitochondrial oxidative phosphorylation of OSCC cells and regulate the expression levels of histone deacetylase 2 and downstream proteins [84]. Triptolide (**51**) significantly inhibits the proliferation, cell cycle arrest, and apoptosis of taxol-resistant SAS/Taxol cells. Kuo et al. [85] found that triptolide inhibits the growth of oral cancer tumour and proliferation of OSCC cells by down-regulating PD-L1 expression. The antibiotic antimycin A (**52**) mediates the apoptosis of OCC CAL27 and Ca9-22 cells by increasing oxidative stress and ROS production [86]. Nitrated [6,6,6] tricycle (**53**)-derived compounds induce apoptosis and DNA damage in OCC by inducing oxidative stress [87]. Meanwhile, pseudolaric acid B (**54**) significantly inhibits the caspase-dependent apoptosis of HN22 cells [88].

**Figure 7:**
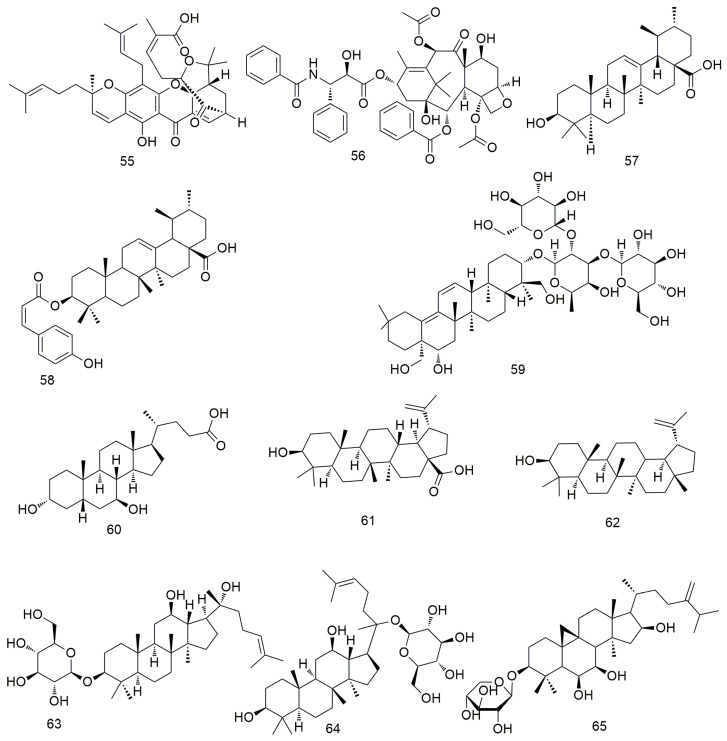
Terpenoid and steroid agents.

Gambogic acid causes the G1 arrest of OSCC cells. In addition, gambogic acid (**55**, Figure 7) can pharmacologically inhibit p38 kinase, significantly reduce haem oxygenase 1 (HO-1) expression, induce caspase cleavage, and promote cell apoptosis [89]. Paclitaxel (**56**) significantly inhibits the activity and proliferation of OCC by increasing the expression of Bim, Bid, MMP-2, and MMP-9. In addition, paclitaxel inhibits the growth of oral cancer cell lines by inhibiting the EGFR signalling pathway [90]. Paclitaxel combined with lupeol inhibits the simulation of hypoxia-induced angiogenesis [91]. Ursolic acid (**57**) induces caspase-dependent cell apoptosis by down-regulating the expression of multiple biomarkers, including Akt/mTOR/NF-xB signalling [92]. It also inhibits angiogenesis by preventing the migration/invasion of Ca922 OCC and blocking the secretion of MMP-2. Cis-3-o-p-hydroxycinnamyl ursolic acid (**58**) inhibits the stagnation of oral cancer cell lines (Ca9-22 and SAS cells) in the G1 phase in a concentration-dependent manner [93]. Additionally, cis-3-O-p-hydroxycinnamoyl ursolic acid triggers the production of intracellular ROS and mediates mitochondrial apoptosis by inducing ROS dependence and p53. Sharifi et al. [94] found that the cytotoxic mechanism of thistle saponins IV (**59**) and IVa may be mediated through the mitochondrial apoptosis pathway and that both saponins can reduce the migration, invasion potential, and metastasis of HN-5 cancer cells. Ursodeoxycholic acid (**60**) induces the apoptosis of cancer cells by promoting the expression of caspase-3/8/9 and reducing the expression of pro-apoptotic proteins [95]. Betulinic acid (**61**) inhibits the proliferation of OSCC cells by regulating ROS and p53 signalling, making it a potential drug for the treatment of oral cancer [96]. Lupeol (**62**) can promote the apoptosis and inhibit the proliferation of OSCC cells by inducing the phosphorylation of EGFR and inhibiting the activation of downstream molecules, such as protein kinase B (or AKT) and NF-κB [91,97]. Zhang et al. [98] found that 20(S)-ginsenoside Rh2 (**63**) induces the apoptosis and inhibits the growth of OCC by inducing G0/G phase arrest and significantly down-regulating the levels of p-Src, p-B-Raf, and p-ERK1/2 proteins. Li et al. [99] found that ginsenoside M1 induces cell apoptosis by increasing the expression of pro-apoptotic protein p53, promoting DNA breakage, and inhibiting the cell cycle. In addition, ginsenoside M1 (**64**) dose-dependently inhibits the colony formation and migration of SAS and OEC-M1 cells and reduces the expression of the transfer-related protein vimentin. Li et al. [100] found that riparsaponin (**65**) inhibits OSCC metastasis by down-regulating the expression of cellular-mesenchymal epithelial transition factor (c-MET), MMP-2, and MMP-9 and by up-regulating the expression of E-cadherin; it also shows significant anti-OSCC activity by inducing mitochondria-mediated apoptosis.

## 7. Other Compounds

**Figure 8:**
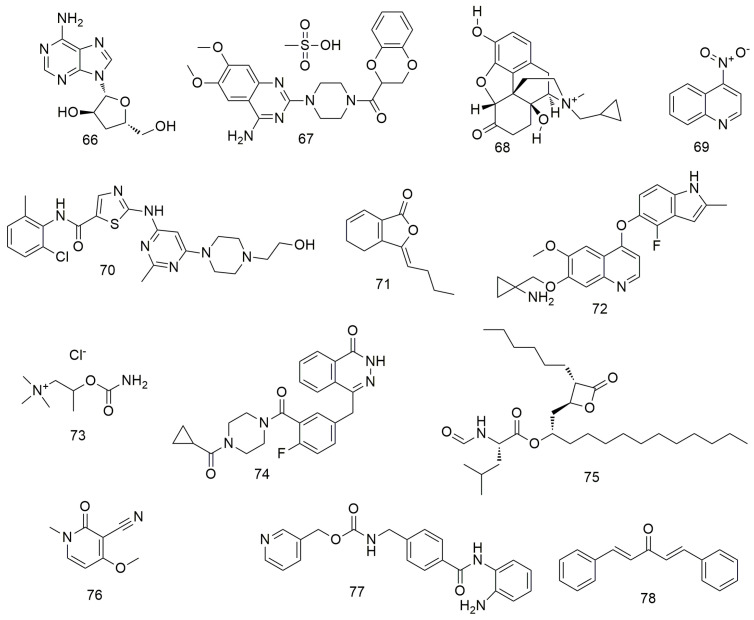
Other agents.

**Figure 9:**
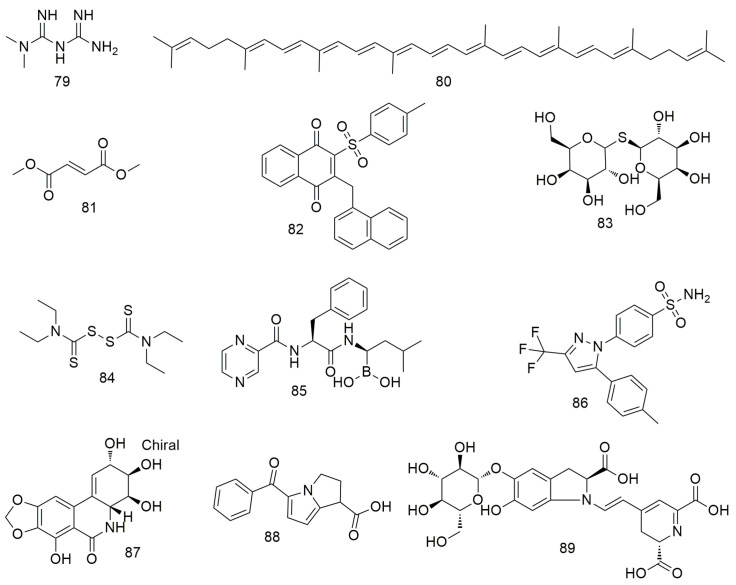
Other agents.

In addition to the above compounds, many other compounds (Figure 8 and Figure 9) induce oral cancer cell cycle arrest, promote cell apoptosis, and inhibit tumour cell metastasis. Cordycepin (**66**) not only regulates the OEC-MI cell cycle but also exerts anti-cancer effects on human OSCC cells when combined with irradiation [101]. Cordycepin (**66**) and IR synergistically induce ATG5 and p21 to inhibit cell proliferation in an autophagy cascade-dependent manner [102]. Li et al. [103] found that doxazosin (**67**) has obvious antioxidant and protective effects on normal cells and can effectively induce the death of oral cancer KB cells by inducing apoptotic signalling. Methylnaltrexone (**68**) strongly inhibits the proliferation, cloning activity, invasion, and migration of FaDu and MDA686Tu cells and inhibits tumour growth in HNSCC-bearing mice [104]. 4-Nitroquinoline (**69**) induces the expression of cancer stem cell (CSC) markers in rat tongue cancer, and candidate CSCs increase in infiltrating areas after SCC [105,106,107]. Dasatinib (**70**) exhibits strong anti-growth, anti-angiogenic, and pro-apoptotic effects on two types of OCC cells (YD-38 and HSC-3) by regulating multiple cell targets and pathways [108]. Ligustilid (**71**) e inhibits the migration of anoxic TW2.6 cells and induces caspase-dependent apoptosis. Hsu et al. [109] demonstrated that ligustilide induces C-MYC-dependent apoptosis in hypoxic oral cancer cell lines (including TW2.6 and OML1) via ER stress signalling. Anlotinib induces G2/M arrest and apoptosis in two oral cancer cell lines, Cal-27 and SCC-25, by targeting the antiangiogenic activity of several tyrosine kinases, including vascular endothelial growth factor receptor, fibroblast growth factor receptor, and platelet-derived growth factor receptor [110]. Olaparib (**74**) treatment significantly reduces the proliferation, migration, invasion, and adhesion of OSS cells. Olaparib inhibits the mRNA expression of markers related to tumourigenesis and EMT, and significantly inhibits tumourigenesis and bone invasion [111]. Orlistat (**75**) induces the apoptosis and cell cycle arrest of HSC-3 cells in the G2/M phase by decreasing the expression of cyclins D1 and E and increasing the phosphorylation of CDK1 [112]. Ricinine (**76**) analogues exert anti-cancer activity by down-regulating protein tyrosine phosphatase (PTP1B) and cyclooxygenase-2 (COX-2) enzymes through highly activated PTP1B protein [113]. Entinostat (**77**) reduces the proliferation and promotes the apoptosis of OSCC cells by causing GO/G1 phase arrest. It can also increase the expression of acetylated histones H3 and H4 and alter the expression of cell cycle-related proteins, such as p21 [114]. Dibenzylideneacetone (**78**) inhibits cell viability and induces apoptosis by degrading specific Spl [115]. It also increases Bax expression, resulting in conformational changes, translocation to the mitochondria, and oligomerisation. In addition, siRNA and miramycin A induce Bax protein expression to increase apoptosis by down-regulating Spl expression.

Metformin (**79**) inhibits the growth and metastasis of oral cancer by down-regulating the expression of Aurora-A and Late SV40 Factor. It also suppresses tumourigenesis in xenotransplantation models [116]. The inhibitory effect of metformin on oral cancer is associated with the decreased expression of OrorA-A. Lycopene (**80**) inhibits the migration and promotes the apoptosis of OSCC cells by blocking the insulin-like growth factor 1 pathway [117]. Dimethyl fumarate (**81**) slows the progression and growth of OSCC by regulating apoptosis and reducing oxidative stress. It also reduces the migration ability of tumour cells by regulating the expression of EMT markers N-cadherin and E-cadherin [118,119]. Tang et al. [120] found that CHW09 (**82**) induces the apoptosis, oxidative stress, and DNA damage of OCC without exerting cytotoxicity to normal cells. Thiodigalactoside (**83**) significantly inhibit the growth, induce the cell cycle arrest and apoptosis, and prevent the angiogenesis of OSCC cells [121]. CuCl_2_ alone or in combination with disulfiram (**84**) significantly reduces ROS levels in the mitochondria of OECM-1 and SG cells [122]. In addition, the binding of disulfiram to Cu^2+^ significantly increases the cytotoxicity of OECM-1 OCC. Bortezomib (**85**) reduces TRAF6 expression via autophagy-mediated lysosomal degradation, which weakens the tumourigenicity of OSCC cells [123]. Celecoxib (**86**) inhibits oral EMT and cell migration by reducing the expression of transcription factors [124]. Narciclasine (**87**) inhibits oral cancer metastasis by regulating ERK pathways and cathepsin B [125]. Ketorolac (**88**) down-regulates DDX3 expression in the human OSCC cell line (H357) and directly inhibits ATP hydrolysis with DDX3 [126]. In addition, treatment with ketoate decreases the number and severity of tongue tumour lesions in a mouse model of carcinogen-induced tongue tumour. Betanin (**89**) can inhibit cell viability, MMP, and inflammation via the NF-kB/PI3K/Akt pathway and increase ROS levels in SCC131 and SCC4 OCC to induce apoptosis [127].

## 8. Conclusions

The incidence and mortality of oral cancer are serious threats to human life and health. This review summarises different types of oral cancer drugs and describes their sources, curative effects, and mechanisms of action, which include inhibiting the proliferation and migration, blocking the cell cycle, and enhancing the autophagy and apoptosis of oral cancer cells (Table 1), but their mechanisms of action are complex and their targets are different. Among many signalling pathways, the AKT/mTOR pathway has been studied the most, which is targeted by 6-gingerol, liquiritigenin, linalool, etc. The anti-oral mechanism of curcumin, phloretin, 6-MITC, and entinostat is through the inhibition of cell cycle. In addition, the promotion of apoptosis and autophagy is also the focus of antitumour small-molecule drug research and development, such as PAC, which has both capabilities. In particular, the IC_50_ value of entinostat is 0.54 µM, which is the best antitumour proliferation activity among these molecular agents. It is worth noting that dasatinib acts as an anti-growth, anti-angiogenesis, and pro-apoptotic agent by regulating multiple targets, including Src, EGFR, STAT-3, STAT-5, PKB, ERK-1/2, S6, eIF-2α, GRP78, caspase-9/3, Mcl-1, and HIF-1α. Therefore, dasatinib can be used as the first choice of anti-oral drugs.

The research on the mechanism of small-molecule anti-oral cancer is still mostly at the characterisation level, and the research on upstream and downstream signal transduction pathways needs to be further deepened. Although there are many studies on small-molecule drugs for oral cancer, there are few clinical studies reported. Therefore, how to improve the availability of drugs, enhance the targeting and accuracy of drugs, so as to better apply in clinical research, is the focus of follow-up research of small-molecule drugs. The application of disulfiram in the treatment of oral cancer provides us with a new idea for the development of antitumour drugs. The new use of old drugs can perfectly avoid the key problems of cancer drug research and development, such as long research and development cycle, high cost, and low success rate. Exploring and understanding the mechanism of action of known active anti-oral cancer compounds is of great significance for the search for new anti-oral cancer drug targets and designing anti-oral cancer drugs with strong effect, good effect, and small side effects.

## Figures and Tables

**Table 1:** Anti-oral cancer small-molecule agents.

No.	Name	Source	Cell Line	Activity (IC_50_)	Target or Signalling Pathway	Reference
1	6-Gingerol	Isolated from ginger	YD10B and Ca9-22	-	AKT/mTOR signalling pathway	[30]
2	Platyphyllenone	Isolated from Alnus nepalensis Isolated from leaves	SCC-9 and SCC-47	-	AKT and c-Jun N-terminal kinase (JNK) pathways	[31]
3	Resveratrol	Isolated from grapes	HSC-2 and HSC-3, HSC-4, Ca9-22, and SAS	-	Autophagy	[32,33]
4	Phloretin	Isolated from plants	SCC-1	12.5 µM	ROS-mediated apoptosis and G0/G1 phase arrest.	[34]
5	Piperlongumine	Isolated from Piper longum	SAS and CGHNC8	-	TNF-α, IL-6, and NF-κB	[35,36]
6	Curcumin	Isolated from ginger	SCC-25	-	Cell cycle arrest	[37]
7	GO-Y078	Synthetic	SCC-9 and HSC-3	<0.5 μM	Caspase-mediated apoptosis	[38]
8	PAC	Synthetic	Ca9-22	3 µM	Apoptosis, autophagy, and oxidative stress	[39]
9	Caffeic acid phenethyl ester	Isolated from propolis	TW2.6, OSF, GNM, TSCCa, SAS and OEC-M1	72.1, 90.6, 101.0, 120.9, 129.7 and 159.2 µM	Apoptosis-related proteins	[40]
10	Rosmarinic acid	Isolated from Rosemarinus officinalis	SCC-15	20–40 µM	Apoptosis and G2/M phase arrest	[41,42]
11 and 12	delta-8- and delta-9-tetrahydrocannabinol	Isolated from cannabis	Ca9-22	13 and 10 μg/mL	Decreased ROS production and increased glutathione and glutathione expression	[43]
13	Pterostilbene	Isolated from rosewood	SAS and OECM-1	-	c-Jun N-terminal kinase (JNK) pathways	[44]
14	Bis(hydroxymethyl)propionate analogs	Synthetic	CAR	32.58 μM	Autophagy	[45]
15	7,8-Dihydroxyflavone	Isolated from plants	HN22 and HSC4	-	Cell cycle arrest and apoptosis	[49]
16	Liquiritigenin	Isolated from liquorice	CAL-27 and SCC-9	-	PI3K/AKT/mTOR pathway	[50]
17	Chrysin	Isolated from bignonia	MC3	-	MAPK/extracellular signalling pathway	[51]
18	Fisetin	Isolated from toxicodendron sylvestre	Ca9-22	-	Wnt, mTOR, and NF-xB signals’ pathway	[52]
19	Quercetin	Isolated from plants	HSC-6 and SCC-9	50 μM	Mitochondrial apoptosis pathway	[53,54]
20	Baicalein	Isolated from Scutellariae Radix	SCC25, CAL27 and HSC3	-	Sp1	[55]
21	luteolin	Isolated from chamomile tea, celery, perilla leaf, and green peppers	SAS and GNM	-	Interleukin-6/signal transduction and transcription 3 signalling	[56]
22	Hydroxygenkwanin	Isolated from Daphne genkwa Sieb. et Zucc.	SAS and OCEM1	-	p21 and endogenous apoptotic pathways	[57]
23	Apigenin	Isolated from fruits and vegetables	SCC-25	-	Cell cycle arrest and apoptosis	[58]
24	Hesperidin	Isolated from fruit of immature citron	HN6	169.53 μM	Programmed Death-Ligand 1 Expression	[59]
25	Luteolin-7-*O*-Glucoside	Isolated from plantain herb	HSC-3, FaDu, and CA9-22	-	Signalling regulates the kinase pathway	[60]
26	Sulforaphane	Synthetic	SCC-9 and SCC-14	-	Cathepsin S	[61,62]
27	Benzyl Isothiocyanate	Isolated from Carica papaya L.	SCC-25	29.80 μM.	Apoptosis	[63]
28	6-MITC	Isolated from Wasabia japonica	SAS and OECM-1	-	G2/M phase	[64]
29	I7447	Semi-synthetic	SAS and OECM-1	10.3 and 13.1 μM.	G2/M phase	[64]
30	I7557	Semi-synthetic	SAS and OECM-1	10.1 and 9.6 μM.	G2/M phase	[64]
31	Chrysophanol	Isolated from rhubarb	FaDu and SAS	9.64 and 12.60 μM.	Cell death, metastasis, and reactivity oxygen production	[65]
32	Aloe emodin	Isolated from Rheum undulatum L.	SCC15	160.7 μM	Apoptosis	[66]
33	Plumbagin	Isolated from Plumbago zeylanica L	CR-SAS	4.379 μM	ROS-mediated endoplasmic reticulum stress and mitochondrial dysfunction	[67]
34	Shikonin	Isolated from alkanet	SCC-25 and HSC-3	-	β-catenin pathway	[69]
35	Acetylshikonin	Isolated from Lithospermum erythrorhizon	YD10B	-	Interleukin-8/matrix metalloproteinase axis	[70]
36	Atorvastatin	Synthetic	HN13	-	VEGF-A after ROS formation	[74]
37–42	Lovastatin, Simvastatin, Fluvastatin, Pravastatin, Pitavastatin, Rosuvastatin	Synthetic	MOC1	-	PD-1	[75]
43	Linalool	Isolated from aromatic camphor	OECM-1	65 µM	PI3K/AKT signalling pathway	[76]
44	Dehydroandrographolide	Isolated from sinularia flexibilis	Ca9-22, SCC-9, OECM-1, CAL 27, OC-2, and HSC-3	-	Apoptosis and oral DNA damage	[78]
45	Coronarin D	Isolated from garland-flower	SCC-9 and SAS	-	JNK1/2 signalling pathway	[79]
46	Costunolide	Isolated from costustoot	YD-10B, YD-38 and Ca9-22 than in YD-9	-	Protein kinase B pathway	[80]
47	4-Carbomethoxyl-10-epigyrosanoldie E	Isolated from sinularia sandensis	Ca9-22 and Cal-27	-	Apoptosis and autophagy	[81]
48	Sinularin	Isolated from S. manaarensis	Ca9-22 and CAL 27	23.5 and 36.6 µM	Oxidative stress-mediated cell G2/M block and apoptosis	[82]
49	Dihydrosinularin	Isolated from S. flexibilis	Ca9-22, OECM-1, CAL 27, and SCC-9	0.39, 0.69, 0.8 and 0.65 mM	Apoptosis and DNA damage	[83]
50	Trichodermin	Isolated from trichoderma viride,	Ca922 and HSC-3	9.65 ± 1.1 µM and 11.49 ± 1.26 µM	Apoptosis, mitochondrial dysfunction, and hdac-2-mediated signalling	[84]
51	Triptolide	Isolated from Thunder God vine	SAS	1.686 nM	Interferon γ modulates the expression of PD-L1 in oral cancer cells in microenvironment	[85]
52	Antimycin A	Isolated from Streptomyces	CAL-27 and Ca9-22	4.72 and 14.85 µM	ROS	[86]
53	Nitrated [6,6,6]Tricycles	Synthetic	Ca9-22, CAL 27, and HSC-3	7.93, 12.46 and 12.46 µM	Apoptosis and DNA damage	[87]
54	Pseudolaric Acid B	Isolated from pseudolarix kaempferi	PAB, HN22	approximately 0.7 µm/mL	Apoptosis	[88]
55	Gambogic Acid	Isolated from garcinia hanburyi and garcinia morella trees	SCC-9 and SAS	-	p38 signals apoptosis in oral cells	[89]
56	Paclitaxel	Isolated from pacific yew tree	tea8113	-	Epidermal growth factor receptor signalling pathways	[90]
57	Ursolic acid	Isolated from bearberry leaf	Ca922 and SCC2095	11.5 and 13.8 μM	Induce apoptosis and autophagy	[92]
58	Cis-3-*O*-p-hydroxycinnamoyl	Isolated from Elaeagnus oldhamii Maxim	Ca9-22 and SAS	24.0 and 17.8 μM	ROS-dependent p53-mediated mitochondrial apoptosis	[93]
59	Buddlejasaponin IV	Isolated from clinopodium umbrosum	HN-5 and HUVEC	19.1 and 18.6	Mitochondrial apoptosis pathway	[94]
60	Ursodeoxycholic Acid	Isolated from gallbladder of Ursus thibetanus	HSC-3	-	Apoptosis	[95]
61	Betulinic Acid	Isolated from plants	KB	-	ROS-regulated p53 signalling	[96]
62	Lupeol	Isolated from plants	SCC131 and SCC084	26.1 and 21.42 lmol	Oncogenic EGFR pathway	[97]
63	20(S)-Ginsenoside Rh2	Isolated from panax ginseng	YD10B and Ca9-22	-	G0/G phase arrest	[98]
64	Ginsenoside M1	Isolated from panax ginseng	OEC-M1	-	Apoptosis	[99]
65	Riparsaponin	Isolated from homonoia riparia	Cal-27, SCC-9 and Detroit 562	-	Apoptosis	[100]
66	Cordycepin	Isolated from cordyceps sinensis	SCC-9, SCC-25, and SAS	-	Autophagy	[101]
67	Doxazosin	Synthetic	KB	-	Modulation of antioxidant and apoptotic pathway	[103]
68	Methylnaltrexone	Synthetic	FaDu and MDA686Tu	-	mu-opioid receptor	[104]
69	4-Nitroquinoline	Synthetic	cancer stem cell	-	Cancer stem cell	[105,106,107]
70	Dasatinib	Synthetic	YD-10B and HSC-3	-	Multi-targeted mechanisms	[108]
71	Z-Ligustilide	Isolated from angelica sinensis	TW2.6 and OML1	-	C-MYC-dependent apoptosis in hypoxic oral cancer cell lines	[109]
72	Anlotinib	Synthetic	Cal-27 and SCC-25	-	Antiangiogenic activity of several tyrosine kinases	[110]
74	Olaparib	Synthetic	EMT	-	mRNA expression of markers related to tumourigenesis and EMT	[111]
75	Orlistat	Synthetic	HSC-3	-	Apoptosis and cell cycle arrest	[112]
76	Ricinine	Isolated from castor bean	SAS	90 µM	PTP1B and COX-2	[113]
77	Entinostat	Synthetic	WSU-HN6 and WSU-HN12	0.54 µM and 23.31 µM	Inhibition of cell cycle	[114]
78	Dibenzylideneacetone	Isolated from Curcuma longa L	HSC-4, HSC-2, YD-10B and SCC-15	-	Specificity protein 1 and Bax	[115]
79	Metformin	Synthetic	SAS, Cal27 and SCC25	-	Malignant behaviour of oral squamous cell carcinoma via a novel signalling involving Late SV40 factor/Aurora-A	[116]
80	Lycopene	Isolated from love apple	CAL-27 and WSU-HN6	0.95 vs. 0.83 mM	IGF1 Pathway	[117]
81	Dimethyl Fumarate	Synthetic	CAL27, HSC-2 and HSC-3	-	Apoptosis, oxidative stress and epithelial–mesenchymal transition	[118,119]
82	CHW09	Synthetic	Ca9-22	40 μg/mL	Apoptosis, oxidative stress, and DNA damage	[120]
83	Thiodigalactoside	Isolated from ilex cornuta	SCC-4, SCC-9 and SCC-25	-	Cell cycle arrest and apoptosis, and prevent the angiogenesis	[121]
84	Disulfiram	Synthetic	OECM-1 and SG	-	Aldehyde dehydrogenase	[122]
85	Bortezomib	Synthetic	SAS	-	Autophagy-mediated TRAF6 oncoprotein degradation	[123]
86	Celecoxib	Synthetic	HSC-3	-	Transcription factors	[124]
87	Narciclasine	Isolated from narcissus	SAS and SCC-47	-	Cathepsin B and extracellular signal-related kinase pathways	[125]
88	Ketorolac	Synthetic	H357	2.6 mM	DDX3	[126]
89	Betanin	Isolated from beets	SCC131 and SCC4	30 μM	NF-κB/PI3K/Akt signalling pathway	[127]
